# IL17/IL17RA as a Novel Signaling Axis Driving Mesenchymal Stem Cell Therapeutic Function in Experimental Autoimmune Encephalomyelitis

**DOI:** 10.3389/fimmu.2018.00802

**Published:** 2018-04-30

**Authors:** Mónica Kurte, Patricia Luz-Crawford, Ana María Vega-Letter, Rafael A. Contreras, Gautier Tejedor, Roberto Elizondo-Vega, Luna Martinez-Viola, Catalina Fernández-O’Ryan, Fernando E. Figueroa, Christian Jorgensen, Farida Djouad, Flavio Carrión

**Affiliations:** ^1^Laboratorio de Inmunología Celular y Molecular, Centro de Investigación Biomédica, Universidad de Los Andes, Santiago, Chile; ^2^Programa de Doctorado en Biomedicina, Facultad de Medicina, Universidad de los Andes, Santiago, Chile; ^3^Programa de Inmunología Traslacional, Facultad de Medicina, Clínica Alemana Universidad del Desarrollo, Santiago, Chile; ^4^IRMB, INSERM, Université de Montpellier, Montpellier, France

**Keywords:** cellular therapy, experimental autoimmune encephalomyelitis, mesenchymal stem cells, IL17, IL17RA

## Abstract

The therapeutic effect of mesenchymal stem cells (MSCs) in multiple sclerosis (MS) and the experimental autoimmune encephalomyelitis (EAE) model has been well described. This effect is, in part, mediated through the inhibition of IL17-producing cells and the generation of regulatory T cells. While proinflammatory cytokines such as IFNγ, TNFα, and IL1β have been shown to enhance MSCs immunosuppressive function, the role of IL17 remains poorly elucidated. The aim of this study was, therefore, to investigate the role of the IL17/IL17R pathway on MSCs immunoregulatory effects focusing on Th17 cell generation *in vitro* and on Th17-mediated EAE pathogenesis *in vivo*. *In vitro*, we showed that the immunosuppressive effect of MSCs on Th17 cell proliferation and differentiation is partially dependent on IL17RA expression. This was associated with a reduced expression level of MSCs immunosuppressive mediators such as VCAM1, ICAM1, and PD-L1 in IL17RA^−/−^ MSCs as compared to wild-type (WT) MSCs. In the EAE model, we demonstrated that while WT MSCs significantly reduced the clinical scores of the disease, IL17RA^−/−^ MSCs injected mice exhibited a clinical worsening of the disease. The disability of IL17RA^−/−^ MSCs to reduce the progression of the disease paralleled the inability of these cells to reduce the frequency of Th17 cells in the draining lymph node of the mice as compared to WT MSCs. Moreover, we showed that the therapeutic effect of MSCs was correlated with the generation of classical Treg bearing the CD4^+^CD25^+^Foxp3^+^ signature in an IL17RA-dependent manner. Our findings reveal a novel role of IL17RA on MSCs immunosuppressive and therapeutic potential in EAE and suggest that the modulation of IL17RA in MSCs could represent a novel method to enhance their therapeutic effect in MS.

## Introduction

The critical role of IL17 in the development of autoimmune and inflammatory disorders such as multiple sclerosis (MS) has been largely investigated (1–4). The pathogenesis of experimental autoimmune encephalomyelitis (EAE), a rodent model of human MS, is characterized by the central nervous system inflammation associated with inflammatory cell infiltration and demyelination (5, 6). An increased production of IL17 has been found in the brain lesions and blood of patients with MS (1, 7). In EAE-induced mice, lymphocytes produce significantly higher levels of IL17 as compared to healthy mice (8). Mice deficient for IL17 are protected from the development of EAE (2) and anti-IL17 treatment reduces the clinical score of EAE mice (9).

The principal receptor of IL17A is IL17RA that also binds IL17F with a lower affinity (10). IL17RA is ubiquitously expressed; however, the main responses to IL17A have been reported to occur in fibroblasts and endothelial cells (11–13). This widespread expression of IL17RA is dynamically modulated on cells according to the stimuli they receive. For instance, in CD8^+^ T cells, cytokines such as IL15 and IL21 increase the expression of IL17RA while phosphoinositide 3-kinase was shown to restrain its expression on T cells (14, 15). In contrast to most interleukin receptors, the expression levels of IL17RA are functionally significant since high levels of IL17RA receptor are required for an effective response (16, 17). Another particularity of IL17RA resides in its capacity to limit the signaling pathway *via* the internalization of its ligand. Indeed, after IL17 binding, it is internalized and removed from the milieu in parallel with a decrease of IL17RA expression level at the cell surface (15).

Mesenchymal stem cells (MSCs) exert potent anti-inflammatory and immunomodulatory effects *via* the suppression or the regulation of different immune cell subset function and proliferation both *in vitro* and *in vivo* (18–21). Using activated mouse CD4^+^ T cells under Th17 skewing conditions *in vitro*, we previously reported that MSCs inhibit the proliferation of Th17 cells as well as their cytokine production (19). Additionally, in an experimental model of arthritis, we have shown that MSCs therapeutic effect is associated with a decreased frequency of pathogenic Th17 cells and the generation of Treg cells bearing the CD4^+^RORγT^+^IL17^+^IL10^+^ signature (22). These immunomodulatory properties of MSCs and their ability to expand *in vitro* without losing their phenotype, multi-lineage, and immunomodulatory potential have generated an increased interest for MSCs as a therapeutic cell of choice for immune-mediated diseases (18,  23). Despite of evidence for a therapeutic potential of MSCs, the underlying mechanisms are not completely understood. MSCs immunoregulatory functions are mediated by the secretion of soluble factors and/or direct cell-to-cell contacts (18, 24, 25). Proinflammatory cytokines such as IFNγ, alone or in combination with TNFα, IL1α, or IL1β have been shown to enhance MSCs immunosuppressive functions (26–28). Indeed, these cytokines alone or in combination trigger the expression of suppressive factors involved in MSC-mediated immunosuppression, such as Programmed Death- Ligand 1 (PD-L1), hepatocyte growth factor, transforming growth factor β1 (TGF-β1), inducible nitric oxide synthase (iNOS), and prostaglandin E2 (PGE2) as well as the expression of adhesion molecules such as VCAM1 and ICAM1 (19, 29–32).

More recently, IL17 has been shown to further enhance the immunosuppressive effect of MSCs induced by IFNγ and TNFα, by promoting the expression of iNOS, revealing an unexpected role of IL17 (33). In accordance with these observations, we have shown that IL17 in presence of IFNγ and TNF-α significantly increases the expression of nitric oxide (NO_2_) and cyclooxygenase 2 expression in MSCs (19). Furthermore, Sivanathan et al. have shown that MSCs pretreated with IL17A enhanced their T cell suppressive effect as well as their capacity to generate regulatory T cells (34). However, inconsistent effects have also been described for IL17-stimulated MSCs. Indeed, IL17 has also been described to reduce the immunosuppressive capacity of olfactory ecto-mesenchymal stem cells (OE-MSCs), mainly by downregulating the levels of inhibitory factors produced by OE-MSCs, such as NO, IL10, TGF-β, as well as PD-L1 (35). Thus, the exact role of IL17 regarding the immunosuppressive effect of MSCs remains to be clarified. Despite the evidence in favor of an enhancing effect of IL17 treatment on MSC-suppressive actions, the involvement and the role of its receptor, IL17RA, has not yet been investigated.

The aim of this study was, therefore, to establish whether the IL17RA is involved in the triggering of the MSC-suppressive effects of Th17 cell function *in vitro*, and in the therapeutic potential of MSCs in the EAE model *in vivo*.

## Materials and Methods

### Mice and Ethical Statement

Females C57BL/6 mice, 10–14 weeks old were purchased from the Central Animal Facility, Instituto de Salud Pública (ISP) in Chile. Animals were housed under standard laboratory conditions and maintained with food and water *ad libitum*. Experimental procedures and protocols were performed according to the US National Institutes of Health Guide for the Care and Use of Laboratory Animals (NIH Publication No. 85-23, revised 1996) and were approved by the Institutional Animal Care and Use Committee of the Universidad de los Andes, Santiago, Chile (Bioethics grant certificate of Universidad de los Andes N°1130444).

IL17RA^−/−^ mice were generated by homologous recombination in ES cells as described (36) and the long bones kindly donated by Wim B. Van Der Berg from Radboud University, Nijmegen were the source for growing these bone marrow-derived cells.

### MSCs Culture and Characterization

Wild-type (WT) and IL17RA^−/−^ MSCs isolation, culture, and characterization were performed as we previously described (20) and according to the criteria described for the International Society of cell therapy (37). For adipogenic differentiation, cells were cultured at 2 × 10^4^ cells/cm^2^ in culture medium supplemented with 1 µM dexamethasone, 60 µM indomethacin, and 10 µg/mL insulin (Sigma-Aldrich, Germany). After 3 weeks, cell differentiation into adipocytes was confirmed by staining of intracellular lipid inclusions with Oil Red O (Sigma-Aldrich). For chondrogenesis, cells were suspended in 15 mL polypropylene conical tubes at the number of 2.5 × 10^5^ cells, centrifuged for 5 min at 600 *g*. The resulting pellets were cultured in 500 µL of the chondrogenic medium containing 10 ng/mL of recombinant TGF-β3 (R&D Systems) for 21 days. Chondrogenic differentiation was confirmed by staining with Safranin O (Merck). Finally, to induce osteogenic differentiation, MSCs were seeded at 3.5 × 10^4^ cells/cm^2^ in culture medium supplemented with 0.1 µM dexamethasone, 0.05 mM ascorbic acid, and 10 mM β-glycerophosphate (Sigma-Aldrich). After 21 days of culture, Alizarin Red staining (Sigma-Aldrich) detected calcium deposits. For the three differentiation processes, medium was changed every 3 days for 3 weeks. MSCs phenotype were assessed by flow cytometry based on the positivity for CD29, CD44, and Sca-1 and in the absence of CD34 and CD45 antigens as we previously described (20).

### Inhibition of IL17RA Expression Using a RNA Interference

Four sequences (GCGCCGAUCAAGAGAAACA; CUGCUUUGAUGUCGUUAAA; CGUAAGCGGUGGCGGUUUU CCGACUGGUUCGAGCGUGA), that in parallel, recognized the mRNA encoding sequence of the IL17RA and the siRNA Control (UAAGGCUAUGAAGAGAUACTT) were obtained from Dharmacon (Dharmacon, England). Briefly, cells were seeded at 70% confluence and transfected with oligofectamine, with IL17RA or control siRNA in opti-MEM^®^ medium following the manufacturer’s instructions. After 12 h, cells were washed and complete DMEM medium (DMEM, 10% FBS, glutamine, and penicillin/streptomicin), and 48 h later, the effectiveness of siRNA was assessed by measuring IL17RA expression by quantitative real-time PCR (qRT-PCR).

### T Cell Proliferation Assay

Splenocytes were isolated from C57BL/6 mice and stained with Carboxyfluorescein diacetate *N*-succinimidyl ester probe (CFSE) or Cell Trace Violet (CTV) (Molecular Probes, USA) according to the manufacturer’s instructions. Then, splenocytes were cultured in the presence or absence of MSCs at a 1:10 ratio (MSCs:splenocytes) in RPMI medium supplemented with 10% heat-inactivated FBS, 2mM l-glutamine, 50 µM mercaptoethanol, 100 U/mL penicillin, and 100 µg/mL streptomycin (Gibco) (complete RPMI media) at 37°C in a 5% CO_2_ incubator and activated with Concanavalin A (ConA) (2 µg/mL) (Sigma Aldrich, USA). After 3 days, cells were harvested and stained with anti-CD3 (BD Pharmingen, USA) and analyzed by FACS.

### Th17 Cells Differentiation and Coculture with MSCs

Purified CD4^+^ T cells were stained with CellTrace™ Violet probe (CTV) (Molecular Probes, USA) and activated with antibodies against CD3/CD28 (BD Pharmingen, USA) and cultured in complete RPMI media as mentioned above. Th17 cells differentiation was induced with 2.5 ng/mL TGF-β1 (PeproTech, USA), 20 ng/mL IL6 (R&D Systems, USA), and 2.5 µg/mL of both anti-IFNγ, and anti-IL4 capture antibodies (BD Pharmingen, USA). WT or IL17RA^−/−^ MSCs were cocultured with CD4^+^ T cells at day 0 of the differentiation process toward Th17 cell lineage at a 1:10 MSCs:T cells ratio. After 5 days of coculture, proliferation and differentiation of T cells was performed by intracellular staining using FACS analysis.

### EAE Induction and MSCs Administration

For EAE induction, 10- to 14-week-old female C57BL/6 mice were injected subcutaneously (s.c.) with 50 µg of MOG_35–55_ peptide (LifeTein LCC, USA) emulsified in complete Freund’s adjuvant (Difco Laboratories, USA), supplemented with heat-inactivated *Mycobacterium tuberculosis* H37RA (Difco Laboratories, USA). At 2 and 48 h, mice also received 300 ng of intraperitoneal (i.p.) Pertussis toxin (Calbiochem, USA). MSCs (1 × 10^6^) were administrated i.p. 5 days after EAE induction and clinical score and animal weight was recorded daily for 22 days. Clinical scores were calculated as previously described (38). Blood samples were collected from mouse tail veins at day 18 after EAE induction and the plasma was obtained after centrifugation (300 × *g*, 20 min).

### Flow Cytometry Analysis

Lymphocytes obtained from the spleen and cocultured with MSCs *in vitro* or from lymph nodes of EAE mice were stimulated *ex vivo* for 4 h with 50 ng/mL phorbolmyristate acetate (Sigma-Aldrich), 1 µg/mL ionomycin (Sigma-Aldrich), and 10 µg/mL brefeldin A (Biolegend, USA). Then, cells were washed in PBS and analyzed for intracellular cytokines. For surface antigen staining, cells were first incubated for 20 min at 4°C in the dark, with antibodies against CD4-PERCP 5.5 and CD25-APC (Miltenyi USA) in the presence of LIVE/DEAD_R_ Fixable near-IR stain (Molecular Probes, USA) to discard dead cells. Then, they were fixed for 30 min at 4°C with the FoxP3 staining buffer set (eBioscience, USA) in order to perform intracellular staining following manufacturer’s instructions. Specific antibodies against Foxp3-PE (Miltenyi, USA), IFNγ (FITC), and IL17-PE (BD Pharmingen, USA) were used.

Mesenchymal stem cells were stimulated with TNFα at 10 ng/mL, IFNγ at 20 ng/mL, and IL17A at 10 ng/mL for 24 h in order to study the phenotype of activated MSCs in response to proinflammatory cytokines. To that end, specific antibodies against VCAM1, ICAM1, and PD-L1 (eBiolegend, USA) were used. Acquisition was performed with a FACS Canto II flow cytometer (BD, Pharmingen) and analyzed with Flow Jo software (Tree Star, USA).

### Cytokine Quantification

Plasma concentrations for a panel of cytokines were measured with the Milliplex mouse Th17 magnetic bead panel Kit (Millipore, USA). Plasma samples were obtained by centrifugation (300 × *g*, 20 min) at day 22 after EAE induction. Plasma samples from 3–4 mice in each group were pooled and analysed by triplicate according to the manufacturer’s instructions. The enzyme-linked immunosorbent assay (ELISA) for GM-CSF and the Enzyme Immunoassay kit for PGE2 (R&D Systems, USA) were used following manufacturer’s instructions with MSCs supernatants from cells activated or not with proinflammatory cytokines. NO_2_ production was quantified in the supernatants of MSCs cocultured with Th17 cells for 5 days, using a modified Griess reagent (Sigma-Aldrich) as previously described by our group (38, 39).

### Reverse Transcription-Polymerase Chain Reaction

Total RNA from cell cultures was isolated using Trizol (Invitrogen) and then treated with DNase I before reverse transcription processing to remove genomic DNA contamination. The reverse transcription was performed according to the manufacturer’s protocol of M-MULV reverse transcriptase (New England BioLab, USA). Briefly, a total of 2 µg RNA from each sample was incubated in a 20 µL reaction volume containing 10× buffer for M-MulV reverse transcriptase (New England BioLab, USA), 20 U RNAse inhibitor (New England BioLab, USA), 1 mM dNTPs, 0.5 µg/µL random primers (Promega), and 200 U M-MuLV reverse transcriptase (New England BioLab, USA) for 5 min at 37°C followed by 60 min at 42°C and 10 min at 70°C. Parallel reactions were performed in the absence of reverse transcriptase to control for the presence of contaminant DNA.

### Real-Time PCR

qRT-PCR reactions were prepared with Hot FIREPol^®^ DNA polymerase (Solis Biodyne, Estonia) in a final volume of 20 µL containing 2 µL cDNA diluted 1:1 and 500 nM primer. PCR reactions were carried out in an Mx3000P QPCR System (Agilent Technologies, USA). The following sets of primers were used: 18S ribosomal RNA (housekeeping gene), sense 5′-GCC CGA AGC GTT TAC TTT GA-3′ and antisense 5′-TTG CGC CGG TCC AAG AAT TT-3′; Ribosomal protein S9 (RS9) (housekeeping gene), sense 5′-GCT GTT GAC GCT AGA CGA GA-3′ and antisense 5′-ATC TTC AGG CCC AGG ATG TA-3′ TGF-β1, sense 5′-CGC TGC TAC TGC AAG TCA GA-3′ and antisense 5′-GTA GCG ATC GAG TGT CCA CG-3′; iNOS, sense 5PCCAG CTG GGC TGT ACA AAC CTT′ and antisense 5′-CAT TGG AAG TGA ACG GTT TCG-3′; IL17RC, sense 5′-GAG TCC CTG CCA GCC ACT T-3′ and antisense 5′-ACT GGA AAT CTT GTG GCT CAT TC; IL17RA, sense 5′-CCC AGT AAT CTC AAA TAC CAC AGT TC-3′ and antisense 5′-CGA TGA GTG TGA TGA GGC CAT A-3′. The thermal cycling conditions consisted of a 10 min denaturation period at 95°C, followed by 40 cycles of denaturation for 30 s at 95°C, annealing for 20 s at 55°C, and extension for 20 s at 72°C. The relative changes in gene expression were calculated by the relative quantification method (2^−ΔΔCt^) (40) and normalized according to the expression of WT MSCs in basal conditions. Differences between two groups were assessed using the Mann–Whitney test. Differences between groups were assessed using Kruskal–Wallis test.

### Statistical Analysis

Statistical analyses were performed using GraphPad Prism 5.0 software (San Diego, CA, USA). Data were expressed as mean ± SD, except for the clinical EAE score, which was expressed as mean. Differences between two groups were analyzed by a non-parametric two-tailed Mann–Whitney test. In order to compare difference of data from more than two groups, the non-parametric Kruskal–Wallis test was used. For the analysis of draining lymph nodes (dLNs), since these data follow the Gaussian distribution, we used ANOVA. All *P-*values <0.05 were considered statistically significant.

## Results

### IL17RA Deficiency Impairs MSCs Immunosuppressive Effect on Th17 Proliferation and Differentiation *In Vitro*

**Figure 1:**
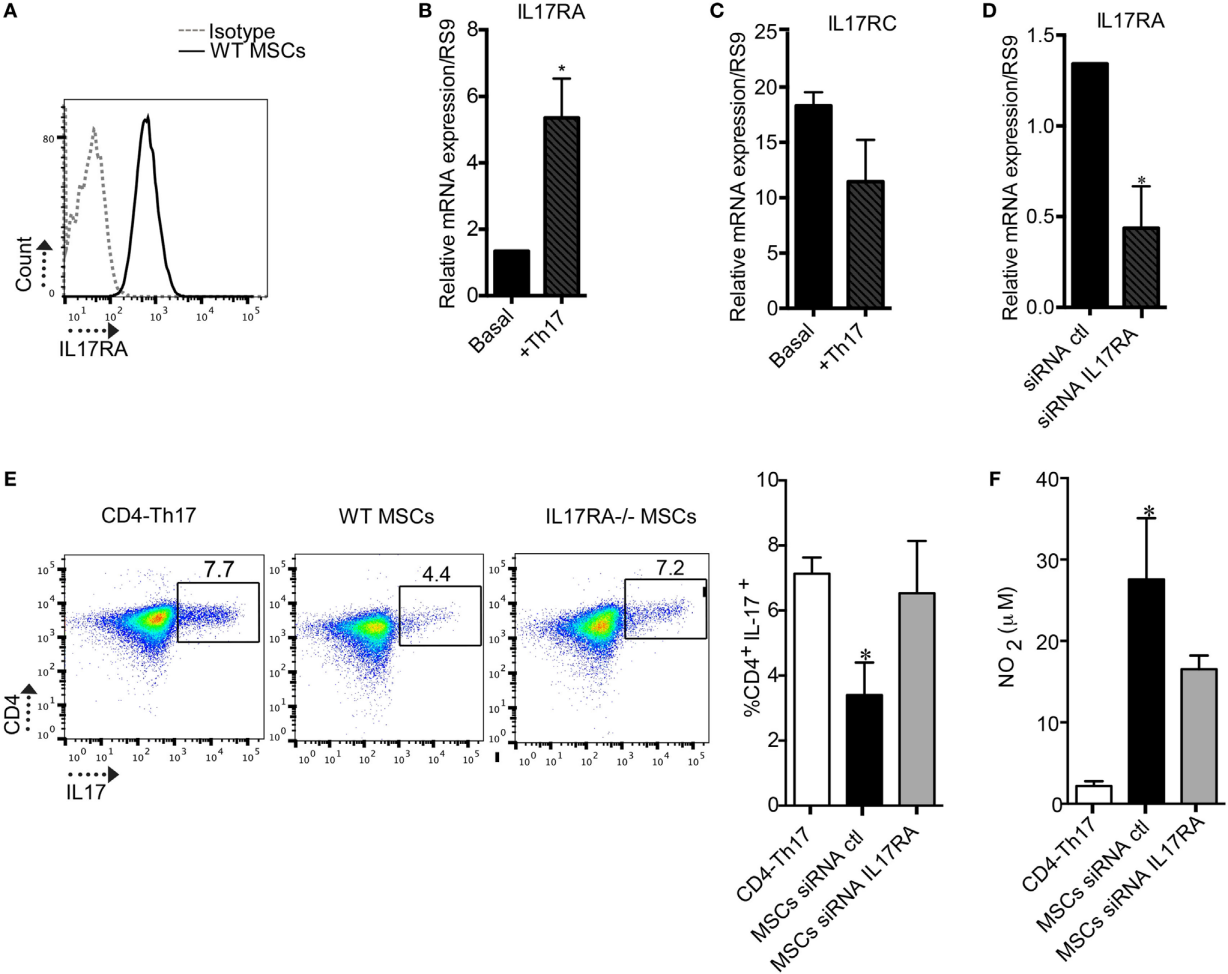
The expression of the IL17RA subunit is critical for mesenchymal stem cells (MSCs)-suppressive effect on Th17 cells. **(A)** Representative histogram of the IL17RA subunit expression in MSCs obtained by FACS. **(B,C)** Quantification of the expression levels of IL17RA and IL17RC subunits in MSCs cultured without or with Th17 cells by quantitative real-time PCR (qRT-PCR). **(D)** Quantification of IL17RA expression level in MSCs after transfection with siRNA control (siRNA ctl) or a siRNA targeting IL17RA (siRNA IL17RA) by qRT-PCR. **(E)** Representative dot plot of CD4^+^IL17^+^ cells (CD4-Th17) analyzed by FACS. Analysis of the percentage of Th17 cells when CD4^+^ T cells undergoing Th17 polarization were cultured alone or with MSCs transfected with either siRNA ctl or siRNA IL17RA during 5 days. **(F)** Quantification of NO_2_ production in the supernatant of Th17 cells cultured alone or with either MSCs siRNA ctl or MSCs siRNA IL17RA. Bars in the plots represent the mean ± SD of *n* = 4 different mice. Statistical differences on cocultures were calculated using the Kruskal–Wallis test. *P < 0.05, **P < 0.005 and for PCR Mann–Whitney test was used.

To determine the role of the IL17/IL17R axis in MSC-mediated immunomodulatory effect, we first evaluated if MSCs express the different subunits of the IL17R (A and C) that bind to the IL17A isoform. MSCs express both subunits of the IL17R (A and C) (Figures 1A–C), but only the subunit A is induced in MSCs when cocultured with Th17 cells (Figures 1B, C). Next, we inhibited the IL17RA subunit using siRNA technology to study the role of this receptor on the suppressive effect of MSCs on Th17 cells (Figure 1D). As compared to MSCs transfected with a control siRNA (siRNA ctl), MSCs silenced for IL17RA (siRNA IL17RA) exhibited a significantly lower inhibitory effect on Th17 cells. Indeed, no significant difference in the percentage of CD4^+^IL17^+^ cells was observed comparing non cocultured Th17 cells with Th17 cells cultured with MSCs that were silenced for IL17RA. However, substantial suppression was observed in the cocultures with control MSCs (Figure 1E). Of note, the loss of MSCs immunosuppressive effect on Th17 cells upon IL17RA silencing was associated with a significant decrease of NO_2_ production. This was one of the main mediators of MSC-suppressive function, in the cultures with IL17RA silenced MSCs when compared to control MSCs (Figure 1F). To further confirm the role of IL17RA on MSCs immunosuppressive properties, we decided to isolate MSCs from the bone marrow of mice deficient for IL17RA (IL17RA^−/−^ MSCs) and their wild-type IL17RA littermates (WT MSCs). First, we studied their phenotype and differentiation capacities into osteoblasts, chondrocytes, and adipocytes *in vitro*. The FACS analysis of MSCs markers showed that both WT MSCs and MSCs negative for IL17RA were positive for CD29, CD44, and Sca-1 and negative for CD34 and CD45 (Figure S1A in Supplementary Material). They also showed multilineage differentiation as revealed by specific staining for adipocytes (Oil Red O), chondrocytes (Safranin O), and osteoblasts (Alizarin Red) (Figure S1B in Supplementary Material). Finally, we showed in IL17RA-/- MSCs did not expressed IL17RA subunit, but expressed IL17RC subunit, as revealed by qRT-PCR (Figure S1C and S1D, respectively in Supplementary Material).

**Figure 2:**
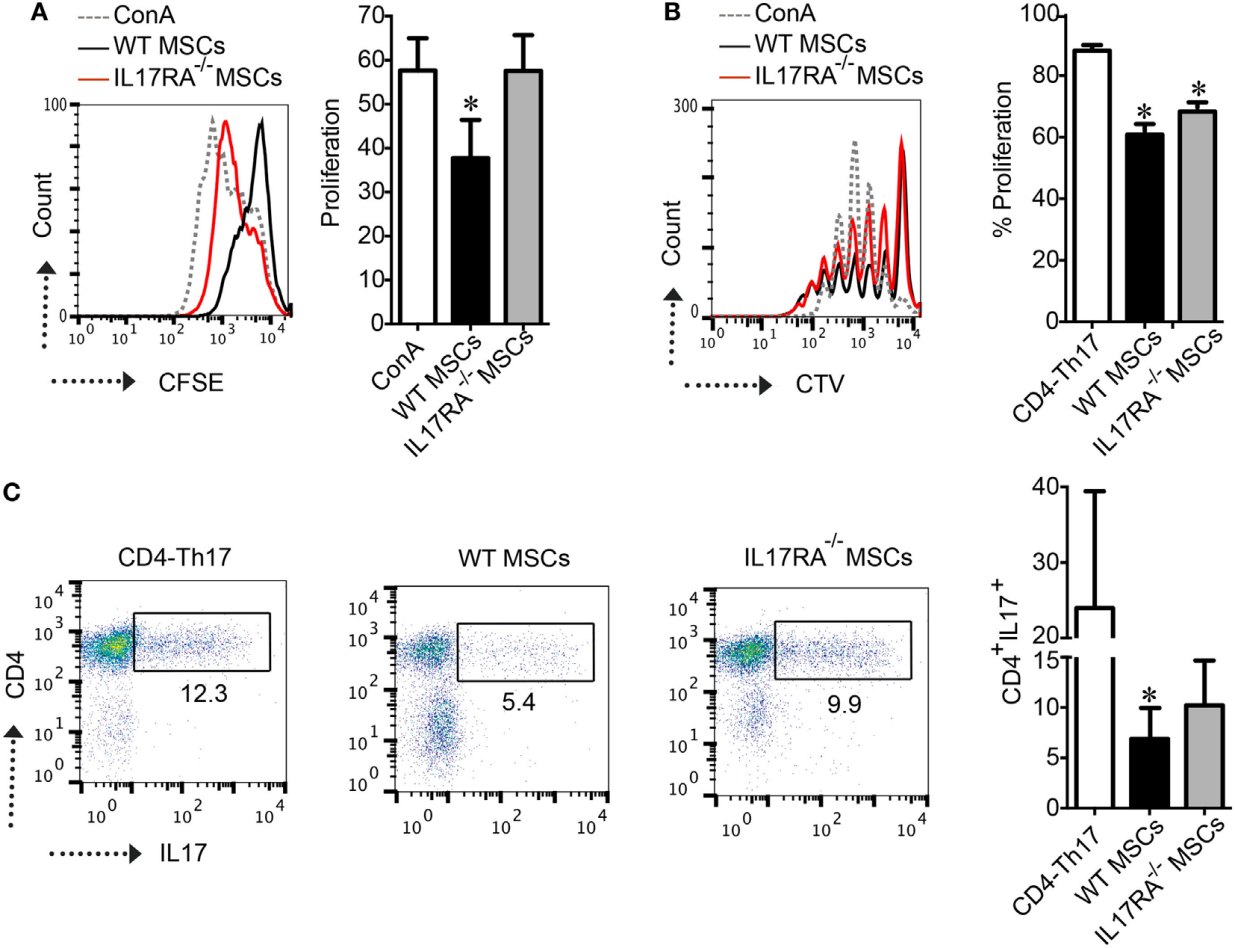
IL17RA deficiency in mesenchymal stem cells (MSCs) impaired their immunosuppressive function. **(A)** CFSE-labeled splenocytes were stimulated with Concanavalin A (ConA) and the proliferation of cells was measured by CFSE dilution by FACS. Results are expressed as the percentage of ConA-induced proliferation. **(B,C)** The effect of wild-type (WT) and IL17RA^−/−^ MSCs on Th17 proliferation **(B)** and differentiation **(C)** was assessed using naïve T-CD4 cells labeled with CTV induced to differentiate into Th17 cells in the absence (CD4-Th17) or presence of WT or IL17RA^−/−^ MSCs at a MSCs:T ratio of 1:10. For T cell proliferation, the percentage of proliferation was measured by CTV dilution by FACS. For Th17 characterization, intracellular detection of IL17 (Th17) was performed by FACS. Bars in the plots represent the mean ± SD of *n* = 4 independent experiments using four different mice each time. Statistical differences were calculated using the Kruskal–Wallis test. *P-*values refer to the condition without MSCs (CD4-Th17). *P < 0.05, **P < 0.01.

Then, we studied, *in vitro*, the immunoregulatory potential of WT and IL17RA^−/−^ MSCs in a proliferation assay with ConA-stimulated splenocytes. Our results showed that after 3 days of coculture, IL17RA^−/−^ MSCs exhibited lower suppressive potential on splenocyte proliferation as compared to WT MSCs (Figure 2A). Finally, we analyzed the effect of IL17RA-deficient MSCs on naïve CD4^+^ T cells cultured in Th17-inducing condition (CD4-Th17) (Figure 2B). Our results showed that the expression of IL17RA in MSCs did not significantly affect the proliferation of CD4-Th17 (Figure 2B). However we observed that IL17RA^−/−^ MSCs, partially, but significantly, impaired their suppressive effect on CD4+ cell on the differentiation into CD4-Th17 cells, when compared to WT MSCs (Figure 2C).These results reveal that the immunomodulatory effects of MSCs on Th17 cell differentiation and proliferation depend, in part, on IL17RA expression.

### The Production of MSC-Derived Immunosuppressive Mediators Depends on IL17RA Expression

**Figure 3:**
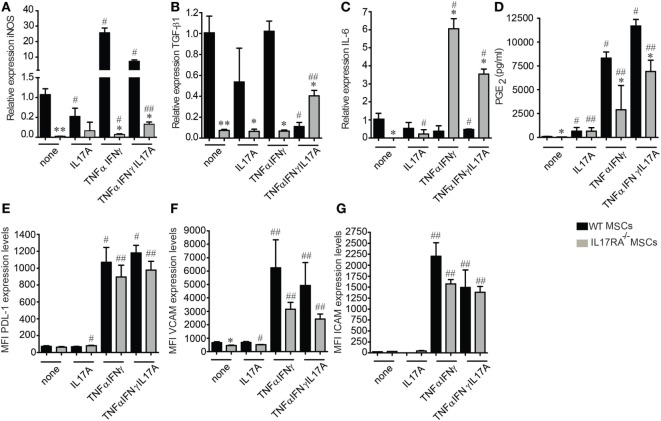
Mesenchymal stem cells (MSCs) deficient for IL17RA produce less suppressive factors associated to MSCs immunomodulatory properties. **(A–C)** Expression profile of inducible nitric oxide synthase (iNOS) **(A)**, transforming growth factor β1 **(B)**, and IL6 **(C)** on wild-type and IL17RA^−/−^ MSCs evaluated by quantitative real-time PCR. The cells were either inactivated (none) or activated (IL17A, TNFα, and IFNγ or TNFα, IFNγ, and IL17A) for 24 h. **(D)** Prostaglandin E2 quantification by enzyme-linked immunosorbent assay from 24 h culture supernatants of MSCs activated or not with IL17A, TNFα, and IFNγ or TNFα, IFNγ, and IL17A. **(E–G)** FACS quantification of PD-L1 **(E)**, VCAM1 **(F)**, and ICAM1 **(G)** on inactivated or activated MSCs (IL17A, TNFα, and IFNγ or TNFα, IFNγ, and IL17A) for 24 h. Statistical differences were calculated using the Kruskal–Wallis test. Each treatment was compared with its respective control (^#^) and between the respective treated condition in wild-type MSCs (*). Mean values of at least three independent experiments. ^*,#^*P* < 0.05, ^**,##^*P* < 0.01.

The enhanced suppressive effect of MSCs has been shown to depend on their activation with proinflammatory molecules such as IFNγ and TNFα. However, the role of IL17, which is mainly produced by Th17 cells has been poorly studied in the context of MSCs immunoregulatory functions. We thus assessed the expression profile of the mediators of MSCs immunosuppressive properties after IFNγ and TNFα activation for 24 h in the presence or absence of IL17A and IL17 alone. For that purpose, GM-CSF, IL6, iNOS, TGF-β1, PGE2, PD-L1, VCAM1, and ICAM1 were measured. qRT-PCR analysis revealed that untreated WT MSCs express significantly higher levels of iNOS and TGF-β1 as compared to IL17RA^−/−^ MSCs (Figures 3A, B). After TNFα and IFNγ activation, the levels of iNOS were significantly increased in both MSCs, although to a greater extent in WT MSCs. TGF-β1 expression level was significantly lower both in non-activated or TNFα and IFNγ activated IL17RA^−/−^ MSCs as compared to WT MSCs (Figure 3B). Interestingly, IL6 expression was significantly increased on the IL17RA^−/−^ MSCs compared to the WT MSCs under TNFα and IFNγ activation in the presence or absence of IL17A (Figure 3C).

Then, we studied the production of PGE2 and GM-CSF by ELISA and found that MSCs deficient for IL17RA produce lower levels of PGE2 than WT MSCs, in basal conditions or after treatment with TNFα and IFNγ with or without IL17A (Figure 3D). No significant difference between the two MSCs was observed in terms of PGE2 secretion under IL17A treatment (Figure 3D). While no significant differences were observed on GM-CSF secretion under any stimulation (data not shown). The increase in the production of PGE2 by the IL17RA^−/−^ MSCs when stimulated with IL17 alone and their high production of TGF-β1 under proinflammatory cytokines plus IL17 could be explained by the presence of the other IL17 receptor subunit RC that could supply in part the role of the IL17RA activity (Figure S1D in Supplementary Material).

Finally, we performed FACS analysis on WT and IL17RA^−/−^ MSCs to evaluate the expression of PD-L1, VCAM1, and ICAM1 on both types of MSCs. Our results showed that the cytokine-induced (TNFα plus IFNγ or TNFα, IFNγ, and IL17A) expression of PD-L1, VCAM1, and ICAM1, triggered by MSCs stimulation was significantly lower with IL17RA^−/−^ MSCs as compared to WT MSCs (Figures 3E–G). Of note, IL17 activation alone did not induce the expression of neither VCAM1, ICAM1, or PD-L1 (Figures 3E–G). The lower expression level of VCAM1, ICAM1, and PD-L1 on IL17RA^−/−^ MSCs as compared to WT MSCs could be associated with their reduced immunosuppressive capacities, since the PD-L1 molecular pathway has been described to be critical for the modulating effect of MSCs on Th17 cells (19). These results indicate that the IL17RA subunit is essential for the expression of key mediators of the MSC-suppressive activity on Th17 cells.

### IL17RA Deficiency Restrains the Effect of MSCs in EAE

**Figure 4:**
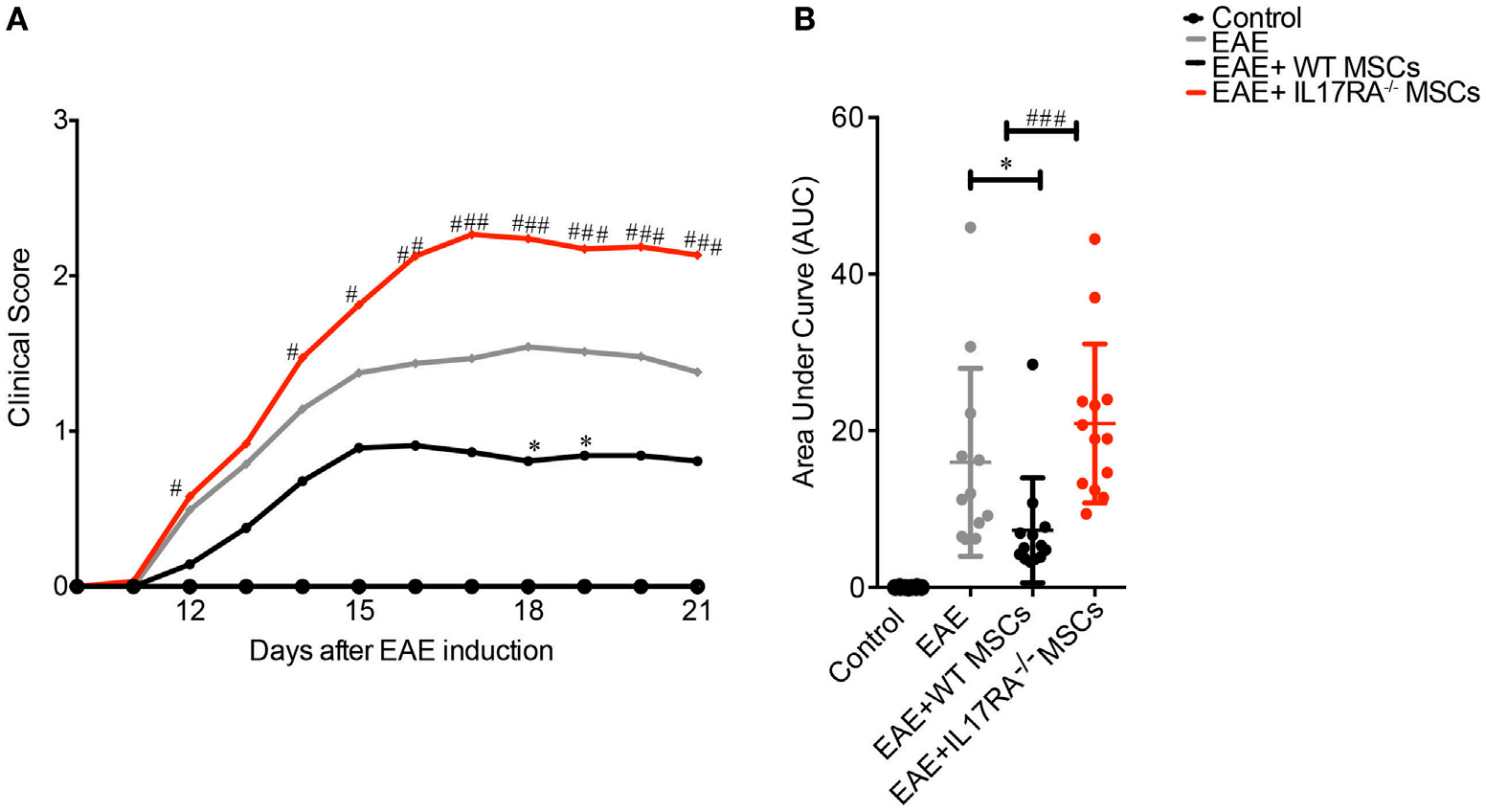
Mesenchymal stem cells (MSCs) deficient for IL17RA failed to prevent experimental autoimmune encephalomyelitis (EAE) development and progression. IL17RA silencing diminish the beneficial effect of MSCs on EAE. **(A)** Daily evaluation of clinical scores were assessed from the day of MSCs injection until the day of euthanasia. **(B)** The area under the curve (AUC) of the clinical scores represented in **(A)** for each treatment was calculated. Line curves of the clinical scores analysis represent the mean and the analysis was performed daily. *Symbol represents the comparison between EAE group compared to EAE animals treated with MSCs [Wild-type (WT) MSC or IL17RA^−/−^]. ^#^Symbol compares the data between EAE animals treated with WT MSCs and IL17RA MSCs. The AUC of clinical scores were calculated for each experimental group. Statistical differences were calculated using Kruskal–Wallis test. Statistical differences were declared significant at *P* < 0.05 level (*P < 0.05, ^###^*P* < 0.001). The results represent two independent experiments considering 16 mice per group at the beginning of the experiment.

To assess the therapeutic potential of IL17RA-deficient MSCs, we induced EAE in C57BL/6 mice with MOG_35–55_ immunization. Four conditions were tested: the control group of healthy mice (Control), the EAE-induced mice (EAE), the EAE mice treated with WT MSCs, and the group treated with IL17RA^−/−^ MSCs. All MSCs were injected i.p. (1 million of cells per mouse) 5 days after EAE induction (Figure 4A). Clinical scores and weight were evaluated daily for 22 days. Consistent with previous reports (38, 41), WT MSCs injected during the first days of EAE induction, before the onset of the disease, induced a significant improvement of the clinical scores in EAE animals (Figure 4A). In contrast, IL17RA^−/−^ MSCs treatment did not exert any beneficial effect on EAE development or progression. Indeed, the administration of IL17RA^−/−^ MSCs induced a worsening of disease characterized by increased clinical scores of EAE (Figures 4A,B). Finally, in order to study the role of the IL17A activation on MSCs, we pre-incubated WT MSCs for 48 h with 30 µg/mL of IL17A, prior to i.p. injection in EAE mice. Our results showed that both non-activated and IL17A-activated MSCs induced a significant decrease of the EAE symptoms. MSCs treated with IL17A exhibited an improved therapeutic effect between days 17 and 24 of disease induction although control MSCs only at days 18 and 24. Area under the curve (AUC) analysis showed that both control MSCs and MSCs treated with IL17 improves the clinical symptoms (Figure S2A in Supplementary Material). Such enhanced therapeutic effect of IL17A-activated MSCs was not detectable in the AUC general analysis (Figure S2B in Supplementary Material).

### IL17RA Deficiency in MSCs Impairs Their Capacity to Inhibit Th17 Cell Response and Regulatory T Cell Generation in the EAE Model

**Figure 5:**
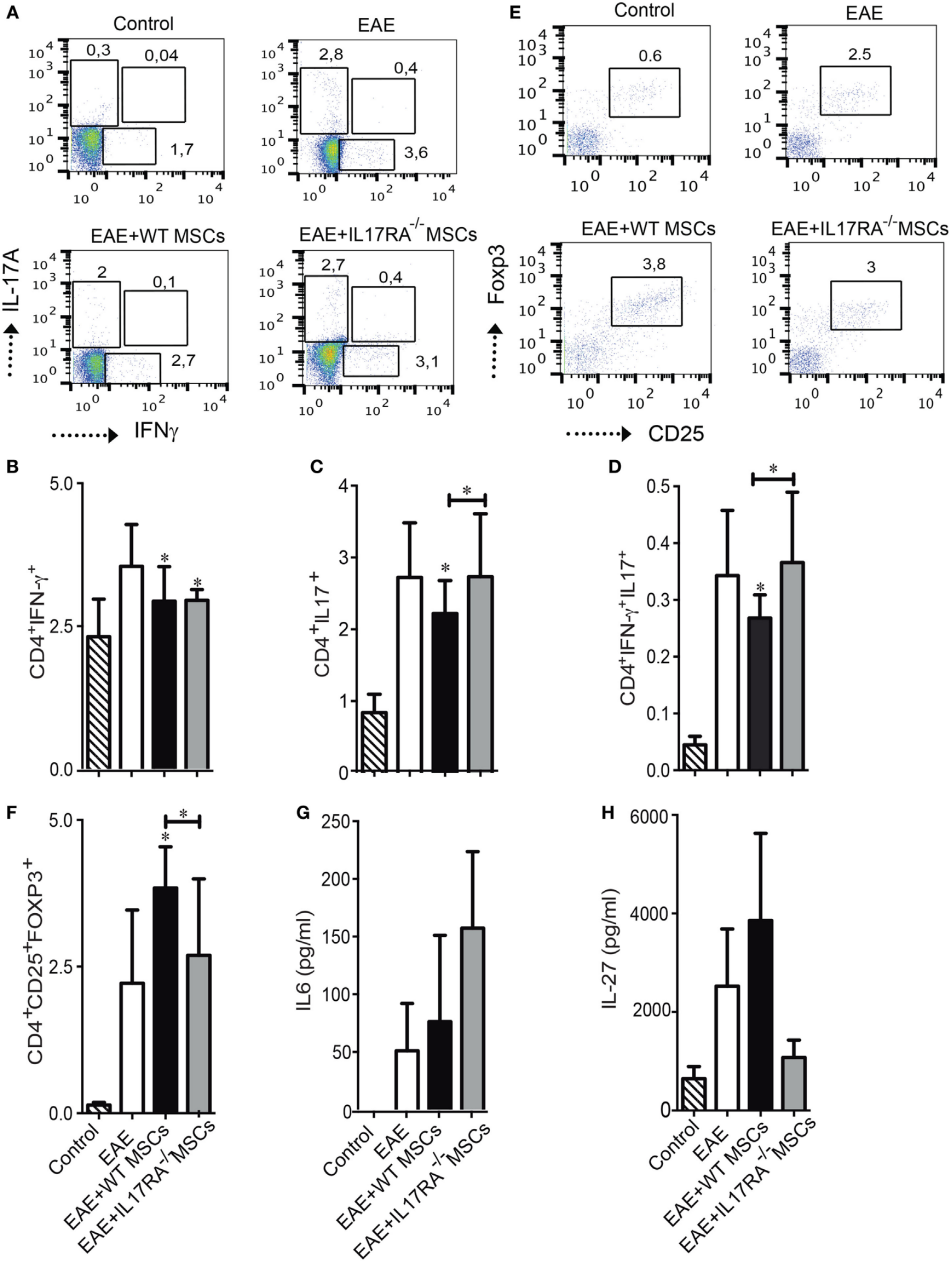
Mesenchymal stem cells deficient for IL17RA failed to reduce the frequency of Th17 cells and to induce Treg generation after administration in experimental autoimmune encephalomyelitis (EAE) mice. **(A)** Representative dot plots of CD4^+^ T cells positive for IL17A and IFNγ in the draining lymph node (dLN) of mice of each experimental group. **(B–D)** Percentage of Th1 cells CD4^+^IFNγ^+^ **(B)**, Th17 cells CD4^+^IL17^+^ **(C)**, and IFNγ^+^ IL17^+^ producing CD4^+^ cells **(D)** were evaluated in the dLN of mice of the different experimental groups at day of euthanasia. **(E)** Representative dot plots of classical Treg cells in the dLN of mice of the different experimental groups. **(F)** Frequency of Treg cells (CD4^+^CD25^+^Foxp3^+^) was assessed in dLN at day of euthanasia. **(G,H)** IL6 and IL27 quantification in the blood of mice 18 days after EAE induction. Bars represent the mean ± SD. Statistical differences were calculated using an ANOVA test. *P < 0.05, **P < 0.005, ***P < 0.0005 compared with EAE group if not indicated by a line. The results represent two independent experiments with a total of 16 mice per experimental group.

Experimental autoimmune encephalomyelitis model is associated with a pathological T cell response leading, in part, to an unbalance of proinflammatory Th cells and classical regulatory T cells (Treg) (42). Therefore, we analyzed the frequency of Th1, Th17, and Treg cells in the dLN of mice of our experimental groups at day of euthanasia. While both WT and IL17RA^−/−^ MSCs were able to reduce the percentage of Th1 cells (CD4^+^IFNγ^+^) (Figures 5A,B), only WT MSCs were able to decrease the percentage of Th17 cells (CD4^+^IL17^+^) (Figure 5C). The percentage of CD4^+^IFNγ^+^IL17^+^ producing cells was also reduced in response to the treatment with WT MSCs (*P < 0.05) (Figure 5D). No effect was observed upon the injection of IL17RA^−/−^ MSCs on this latter T cell subset. Since, the balance between Th17 cells and Treg cells is crucial for the progression of the disease, we also studied the percentage of classical Treg cells. Our results showed that only WT MSCs injection was able to increase the percentage of classical Treg CD4^+^CD25^high^Foxp3^+^ (*P < 0.05) (Figures 5E,F).

In order to evaluate the effect of MSCs injection on cytokine plasma levels in EAE mice, we used a Milliplex mouse magnetic bead panel Kit to measure a large panel of cytokines produced by Th1, Th17, and Treg cells. We found that the plasma levels of IL6 and IL27 in the EAE mice were different (Figures 5G,H). Although no significant difference was detected between the groups, we found that IL6 concentration in the plasma of mice treated with IL17RA^−/−^ MSCs tended to be higher than in control, EAE, and WT MSC-treated mice (Figure 5G). On the other hand, we found that IL27 concentration tended to be higher in the plasma of mice treated with WT MSCs as compared to the mice of the three other groups (Figure 5H). We detected no differences regarding other cytokines such as IFNγ, TNFα, TNFβ, IL12P40, IL10, IL17A, IL17F, and IL17E (data not shown).

## Discussion

In the present study, we evaluated the role of the IL17/L17RA pathway in the immunomodulatory capacities of MSCs *in vitro* and in a Th17-mediated disease model such as EAE. Our results demonstrated both that the expression of the IL17RA subunit by MSCs is crucial for their Th17 suppressive functions and that the IL17/IL17RA axis contributes significantly to the activation of MSCs.

It is well known that the activation or “priming” of MSCs with proinflammatory cytokines is required to trigger their immunosuppressive function. In particular, it has been widely shown that IFNγ together with TNFα, IL1α, or IL1β triggers the expression of critical suppressive factors that mediate MSCs immunomodulatory properties (29). More recently, the effects of IL17A on MSCs function have also been investigated. IL17A strongly promotes the proliferation and the generation of colony-forming unit-fibroblast of both human and murine MSCs (43). Furthermore, MSCs treatment with IL17A significantly modulates their migration, motility, and osteoblasts differentiation potential, confirming an important role of IL17A on MSCs function (44). Regarding the effect of IL17A on MSC-mediated immunoregulatory properties, conflicting results have been published. IL17 alone or together with IFNγ and TNFα on MSCs has been shown to have a positive effect on their immunosuppressive functions since IL17A addition considerably enhances their immunosuppressive potential (33, 34). However, it has been shown that IL17 could also reduce the suppressive capacity of OE-MSCs, mainly by downregulating their production of immunosuppressive factors including NO_2_, IL10, TGF-β1, as well as PD-L1 (35). In the present study, we demonstrate that the IL17/IL17RA axis plays a key role on MSCs immunomodulatory properties since the inhibition of IL17A receptor significantly impairs the capacity of MSCs to inhibit the proliferation and generation of proinflammatory Th17 cells. This loss of MSCs immunoregulatory function in the presence of IL17RA deficiency is associated with a reduced capacity of such MSCs to produce many of the well-established mediators of MSC-associated immunosuppressive properties.

In addition, in our study, we have evaluated the effect of the IL17/L17RA axis in a Th17 immune-mediated disease such as the EAE model (45). It has been widely demonstrated that MSCs exert a significant therapeutic effect in EAE (38, 46, 47). Thus, in order to validate our *in vitro* results, we compared the treatment of EAE mice with either WT or IL17RA^−/−^ MSCs. We showed that while WT MSCs significantly improved EAE symptoms, MSCs deficient for IL17RA exacerbated the progression of disease, providing the first evidence that the expression of the IL17RA subunit is critical for the therapeutic effect of MSCs in EAE. Moreover, when we pretreated MSCs with IL17A prior to their injection into diseased mice, we enhanced their therapeutic effect. Further analysis of the treated and control EAE mice indicated that the IL17RA deficiency of MSCs would suppresses their capacity to restrain pathogenic Th17 cells as well as their ability to induce Tregs in regional lymph nodes. Of note, while IL17RA^−/−^ MSCs administration exacerbated the clinical symptoms of EAE mice, we did not observe any increase in the number of pro-inflammatory T cells (Th1 or Th17), or a decrease in the number of regulatory T cells as compared to untreated mice. This might be because the analysis of these different T cell subsets was performed at day 22, day of EAE induction. This might have been distant to the time-point at which we observed the changes in the clinical score associated with each MSCs treatment.

The lack of IL17RA^−/−^ MSCs therapeutic effect could be in part due to the reduced level of expression of VCAM1, ICAM1, and PD-L1 on IL17RA^−/−^ MSCs as opposed to WT MSCs. This could result in an impaired capacity to migrate and/or interact with pro-inflammatory Th17 cells and thus to suppress their activity or eventual Treg conversion. Moreover, in the absence of an inflammatory environment (characterized by low levels of pro-inflammatory cytokines), the MSCs, sensor, and switcher of inflammation (48), adopt a pro-inflammatory phenotype and activate T cell responses through the release of chemokines that recruit lymphocytes to the inflammation site (29, 49). We could thus reasonably conceive that IL17RA^−/−^ MSCs are less responsive to inflammatory cytokines and thus more prone to adopt a pro-inflammatory phenotype, which could explain that, in the EAE model, they exacerbate the symptoms of the disease. Finally, this pro-inflammatory phenotype adopted by IL17RA^−/−^ MSCs, derived from a global (not cell-type specific) knockout, could also reflect the altered immunological environment in which they have developed, i.e., in the presence of immune cells lacking the IL17 receptor.

## Conclusion

These results demonstrate that the expression of IL17RA on MSCs allows them to efficiently respond to Th17-related proinflammatory microenvironment enhancing their immunosuppressive properties on Th17 cells *in vitro* and, *in vivo*, in Th17-mediated disorders. Altogether, our data propose that the IL17/IL17RA axis plays a significant role in the process of MSCs “licensing” that is at the core of their immunosuppressive and therapeutic potential.

## Ethics Statement

Females C57BL/6 mice, 10–14 weeks old were purchased from the Central Animal Facility, Instituto de Salud Pública (ISP) in Chile. Animals were housed under standard laboratory conditions and maintained with food and water *ad libitum*. Experimental procedures and protocols were performed according to the US National Institutes of Health Guide for the Care and Use of Laboratory Animals (NIH Publication No. 85-23, revised 1996) and were approved by the Institutional Animal Care and Use Committee of the Universidad de los Andes, Santiago, Chile (Bioethics grant certificate of Universidad de los Andes N°1130444). IL17RA^−/−^ mice were generated by homologous recombination in ES cells as described (36) and the long bones kindly donated by Wim B. Van Der Berg from Radboud University, Nijmegen were the source for growing these bone marrow-derived cells. Bioethics grant certificate of Universidad de los Andes N°1130444.

## Author Contributions

This study was designed by PL-C, MK, FD, and FC with the input of CJ and FF. PL-C, MK, AV-L, RC, RE-V, CF-O, LM-V, and FD, performed experiments and analyzed the results. PL-C, MK, FD, AV-L, and FC wrote the manuscript with inputs from FF and CJ. All authors read and approved the final manuscript.

## Conflict of Interest Statement

The authors declare that the research was conducted in the absence of any commercial or financial relationships that could be construed as a potential conflict of interest.

## Supplementary Material

Figure S1 Phenotypic characterization and differentiation potential of wild-type (WT) and L17RA^−/−^ mesenchymal stem cells (MSCs). **(A)** WT and IL17RA^−/−^ MSCs were stained with specific antibodies against CD45, CD34, CD29, CD44, and Sca-1 and analyzed by flow cytometry. Histograms are representative of at least three independent experiments. **(B)** Differentiation potential of WT and IL17RA^−/−^ MSCs. The differentiation potential to the three lineages was analyzed by specific staining for adipocytes (oil red O), chondrocytes (Safranin O), and osteoblasts (Alizarin Red). **(C)** IL17RA expression level was evaluated by quantitative real-time PCR (qRT-PCR) in both WT and IL17 RA^−/−^ MSCs. **(D)** IL17RC expression level was evaluated by qRT-PCR in both WT and IL17 RA^−/−^ MSCs. Bars represent the mean ± SD. Statistical differences were calculated using the Mann–Whitney test. **P* < 0.05.

Figure S2 IL17 mesenchymal stem cells (MSCs) pretreatment increases MSCs therapeutic potential in experimental autoimmune encephalomyelitis (EAE). **(A)** Daily evaluation of clinical score was performed from the day of MSCs administration until euthanasia. **(B)** The area under the curve (AUC) of the clinical score for each treatment was calculated and compared. Line curves of the clinical score analysis represent the mean and the analysis was performed daily. Bars represent the mean ± SD. *Symbol represents the comparison between EAE group compared to EAE animals treated with MSCs (WT MSC or IL17RA^−/−^). The AUC of clinical score was calculated for each experimental group. Statistical differences were calculated using Kruskal–Wallis test. Statistical differences were declared significant at *P* < 0.05 level (**P* < 0.05, ***P* < 0.01). The results represent two independent experiments considering 12–20 mice per experimental group.
