# Supplementary figures and images for: IL17/IL17RA as a Novel Signaling Axis Driving Mesenchymal Stem Cell Therapeutic Function in Experimental Autoimmune Encephalomyelitis
Source: Front Immunol. 2018 Apr 30;9:802. doi: 10.3389/fimmu.2018.00802 (PMC5936796; doi:10.3389/fimmu.2018.00802)

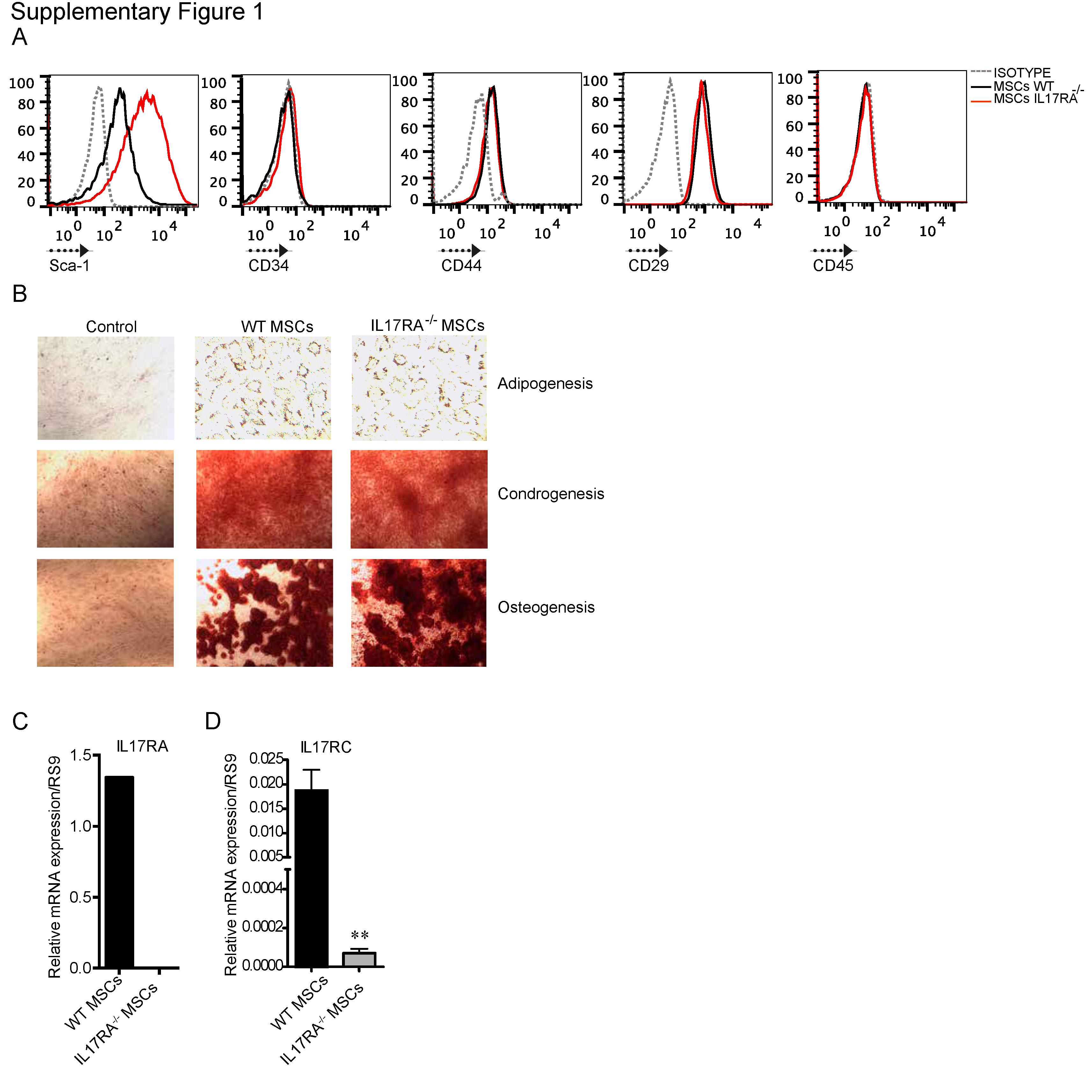

Supplement: Figure S1 — Phenotypic characterization and differentiation potential of wild-type (WT) and L17RA−/− mesenchymal stem cells (MSCs). (A) WT and IL17RA−/− MSCs were stained with specific antibodies against CD45, CD34, CD29, CD44, and Sca-1 and analyzed by flow cytometry. Histograms are representative of at least three independent experiments. (B) Differentiation potential of WT and IL17RA−/− MSCs. The differentiation potential to the three lineages was analyzed by specific staining for adipocytes (oil red O), chondrocytes (Safranin O), and osteoblasts (Alizarin Red). (C) IL17RA expression level was evaluated by quantitative real-time PCR (qRT-PCR) in both WT and IL17 RA−/− MSCs. (D) IL17RC expression level was evaluated by qRT-PCR in both WT and IL17 RA−/− MSCs. Bars represent the mean ± SD. Statistical differences were calculated using the Mann–Whitney test. *P < 0.05. [file Image_1.tif]

Supplementary Figure 2

A

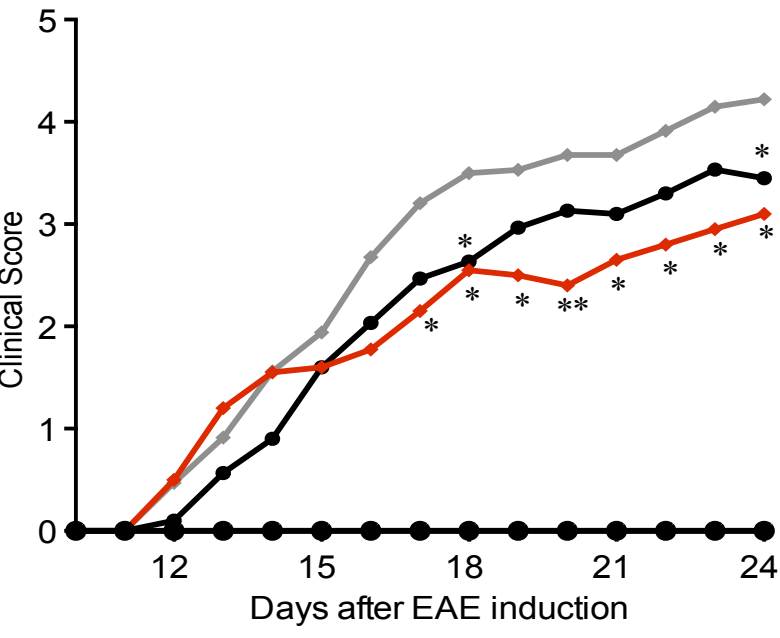

B

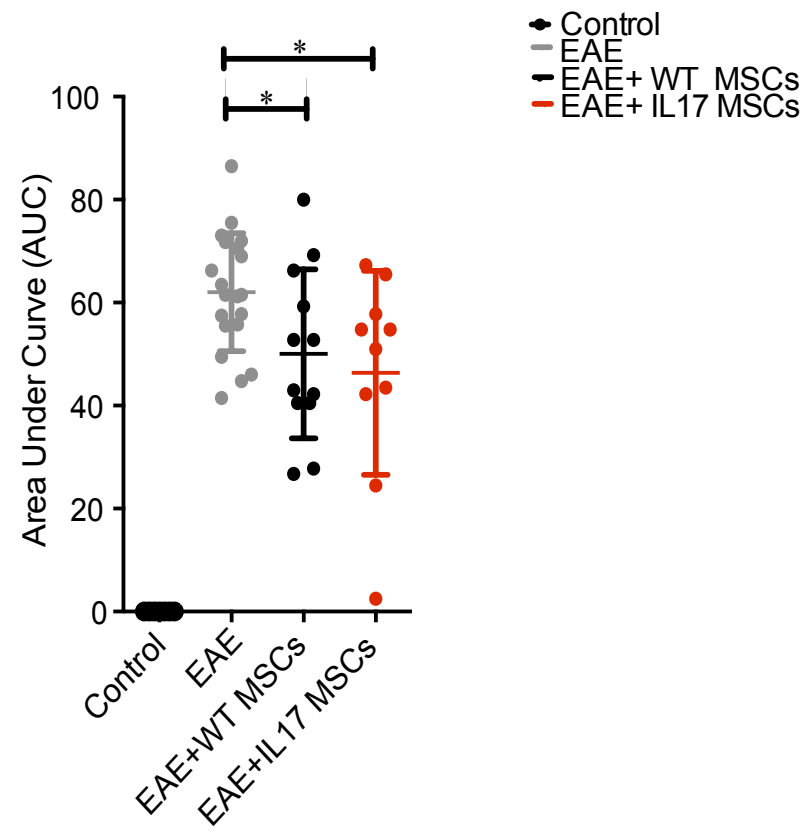

Supplement: Figure S2 — IL17 mesenchymal stem cells (MSCs) pretreatment increases MSCs therapeutic potential in experimental autoimmune encephalomyelitis (EAE). (A) Daily evaluation of clinical score was performed from the day of MSCs administration until euthanasia. (B) The area under the curve (AUC) of the clinical score for each treatment was calculated and compared. Line curves of the clinical score analysis represent the mean and the analysis was performed daily. Bars represent the mean ± SD. *Symbol represents the comparison between EAE group compared to EAE animals treated with MSCs (WT MSC or IL17RA−/−). The AUC of clinical score was calculated for each experimental group. Statistical differences were calculated using Kruskal–Wallis test. Statistical differences were declared significant at P < 0.05 level (*P < 0.05, **P < 0.01). The results represent two independent experiments considering 12–20 mice per experimental group. [file Image_2.pdf]
